# Increased efficacy of metformin corresponds to differential metabolic effects in the ovarian tumors from obese *versus* lean mice

**DOI:** 10.18632/oncotarget.20754

**Published:** 2017-09-08

**Authors:** Jianjun Han, Weiya Z. Wysham, Yan Zhong, Hui Guo, Lu Zhang, Kim M. Malloy, Hallum K. Dickens, Gene Huh, Douglas Lee, Liza Makowski, Chunxiao Zhou, Victoria L. Bae-Jump

**Affiliations:** ^1^ Department of Surgical Oncology, Shandong Cancer Hospital and Institute, Shandong Academy of Medical Sciences, Jinan, Postdoctoral Mobile Station of Tianjin Medical University, Tianjin, P.R. China; ^2^ Division of Gynecologic Oncology, Department of Obstetrics and Gynecology, University of North Carolina, Chapel Hill, NC, USA; ^3^ Legacy Medical Group, Gynecologic Oncology, Portland, OR, USA; ^4^ Department of Gynecologic Oncology, Linyi Cancer Hospital, Linyi, Shandong, P.R. China; ^5^ Department of Gynecologic Oncology, Shandong Cancer Hospital & Institute, Jinan, P.R. China; ^6^ Virginia Tech/Carilion Clinic, Department of Obstetrics and Gynecology, Blacksburg, VA, USA; ^7^ Seoul National University College of Medicine, Seoul, South Korea; ^8^ Omic Insight, Durham, NC, USA; ^9^ Department of Nutrition, University of North Carolina, Chapel Hill, NC, USA; ^10^ Lineberger Comprehensive Cancer Center, University of North Carolina, Chapel Hill, NC, USA

**Keywords:** metformin, metabolism, ovarian cancer, mTOR pathway, obesity

## Abstract

Obesity is a significant risk factor for ovarian cancer (OC) and associated with worse outcomes for this disease. We assessed the anti-tumorigenic effects of metformin in human OC cell lines and a genetically engineered mouse model of high grade serous OC under obese and lean conditions. Metformin potently inhibited growth in a dose-dependent manner in all four human OC cell lines through AMPK/mTOR pathways. Treatment with metformin resulted in G1 arrest, induction of apoptosis, reduction of invasion and decreased hTERT expression. In the K18-gT_121_^+/-^; p53^fl/^ ^fl^; Brca1^fl/fl^ (KpB) mouse model, metformin inhibited tumor growth in both lean and obese mice. However, in the obese mice, metformin decreased tumor growth by 60%, whereas tumor growth was only decreased by 32% in the lean mice (p=0.003) compared to vehicle-treated mice. The ovarian tumors from obese mice had evidence of impaired mitochondrial complex 2 function and energy supplied by omega fatty acid oxidation rather than glycolysis as compared to lean mice, as assessed by metabolomic profiling. The improved efficacy of metformin in obesity corresponded with inhibition of mitochondrial complex 1 and fatty acid oxidation, and stimulation of glycolysis in only the OCs of obese *versus* lean mice. In conclusion, metformin had anti-tumorigenic effects in OC cell lines and the KpB OC pre-clinical mouse model, with increased efficacy in obese *versus* lean mice. Detected metabolic changes may underlie why ovarian tumors in obese mice have heightened susceptibility to metformin.

## INTRODUCTION

Epithelial ovarian cancer (OC) is the leading cause of death from gynecological malignancies and the fifth leading cause of cancer-related death among women in the United States [1]. Due to the asymptomatic nature of early stage disease, women are diagnosed with advanced stage disease in more than 70% of cases, with an overall 5-year survival of only 30-40% [2, 3]. Obesity is an important risk factor for OC and is associated with worse outcomes for this disease [4–18], with up to a 1.5 fold increased risk of death [10]. Therefore, a targeted metabolic approach to the treatment of OC may provide a novel strategy to improve outcomes for this invariably lethal disease.

Excess fuel storage in obesity culminates in stimulated growth factor signaling *via* the insulin/insulin-like growth factor (IGF-1) axis, and can lead to a nutrient-saturated environment with high levels of glucose and other nutrients [19]. Hyperinsulinemia, IGF-1 and IGF-1 receptor (IGF-1R) levels are known to be important in OC development and progression [20–23] through interactions with the downstream PI3K/Akt/mTOR pathway [24–27]. Components of this pathway are often mutated, amplified, or aberrantly expressed in OCs, and are currently being targeted for OC treatment [28–34]. Given the interplay between obesity, insulin/glucose signaling and OC, we hypothesized that obesity creates a unique environment contributing to the generation of tumors that are metabolically distinct from those developing in a “lean” host milieu. In support of this hypothesis, we have previously reported that diet-induced obesity (DIO) in the K18-gT_121_^+/-^; p53^fl/fl^; Brca1^fl/fl^ (KpB) OC mouse model results in a tripling of tumor growth [35]. Furthermore, significant genomic and metabolic differences were demonstrated between ovarian tumors that arose in obese *versus* lean mice [35]. Thus, obesity-driven tumors may have metabolic vulnerabilities that could be targetable for treatment with agents such as metformin.

Metformin is an effective, well-tolerated and inexpensive medication for improving hyperglycemia in the treatment of type 2 diabetes [36]. Epidemiological evidence suggests that metformin lowers cancer risk and reduces cancer incidence and deaths among diabetic patients [37–40], including OC [41–44]. This has led to the hypothesis that metformin could be used for cancer treatment and prevention.

Metformin may have both indirect and direct effects on tumor growth [45]. Its indirect effects are postulated to be due to a reduction in circulating glucose and insulin levels in the host *via* inhibition of gluconeogenesis in the liver, and subsequent decreased growth factor stimulation in tumor cells. On the cellular or direct level, metformin inhibits mitochondrial respiratory complex I, leading to suppression of tricarboxylic acid (TCA) cycle flux, interrupted oxidative phosphorylation, and decreased mitochondrial ATP production [45–48]. The resulting cellular energetic stress from inhibition of complex I raises the AMP/ATP ratio, resulting in increased AMPK signaling and stimulated glycolysis and fatty acid oxidation. AMPK is a central regulator of multiple signaling pathways that control cellular proliferation and metabolism, including inhibition of the mTOR pathway (i.e. specifically mTORC1) [45]. In addition, metformin inhibits the mTOR pathway *via* AMPK-independent mechanisms, potentially through its effects on the Ragulator complex (Rag GTPase) and REDD1 upregulation or *via* enhanced PRAS40 binding to RAPTOR [45, 49–52]. Thus, a drug such as metformin that decreases circulating glucose and insulin levels, inhibits mitochondrial complex I, and disrupts the mTOR pathway may be useful in obesity- and mTOR pathway-driven cancers, such as OC. Given this, our goal was to evaluate the anti-tumorigenic effects of metformin in human OC cell lines and the KpB genetically engineered mouse model of high grade serous OC under both lean and obese conditions.

## RESULTS

### Metformin inhibited cell proliferation in OC cells

**Figure 1:**
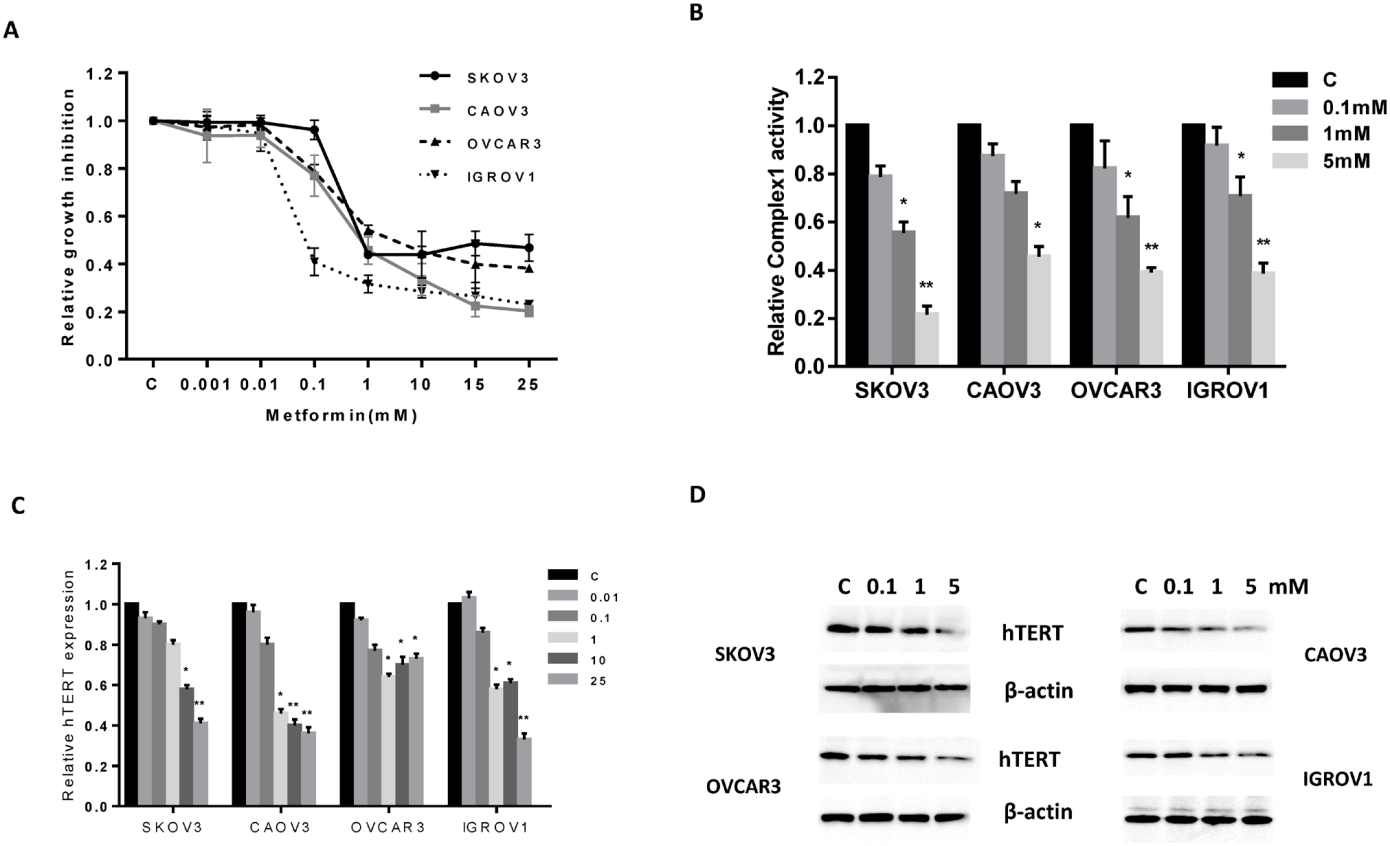
Effect of metformin on proliferation of human OC cells The CAOV3, IGROV1, OVCAR3 and SKOV3 cells were cultured in the presence of varying concentrations of metformin (0.001 – 25 mM) for 72 h. Cell proliferation was determined by MTT assay. Metformin significantly inhibited cell proliferation in all four cell lines **(A)**. Metformin significantly reduced cellular complex 1 activity in a dose-dependent manner after 24 h of treatment **(B)**. Metformin decreased hTERT mRNA expression and hTERT protein expression in a dose dependent manner **(C** and **D)**. The results are shown as the mean ± SE of triplicate samples and are representative of three independent experiments. *p<0.05, **p<0.01.

In all four OC cell lines, treatment with metformin resulted in the inhibition of cell proliferation in a dose-dependent manner compared to vehicle-treated controls. The mean IC50 for the OC cell lines after 72 h of treatment was between 0.1 and 13 mM (Figure 1A). Of all the OC cell lines tested, the IGROV1 cells were found to be the most sensitive to metformin.

The primary cellular target of metformin is mitochondrial respiratory-chain complex 1. To determine if metformin treatment inhibited complex 1 activity in the OC cell lines, cells were treated with increasing concentrations of metformin for 24 h and then the activity of complex 1 was measured by ELISA assay. Metformin significantly inhibited cellular complex 1 activity in a dose-dependent manner (p<0.5) (Figure 1B).

Given that hTERT expression is thought to be a sensitive marker of telomerase function as well as cell proliferation [53], we evaluated hTERT mRNA and protein expression in all four OC cell lines. Real-time RT-PCR was used to quantify hTERT mRNA expression, and Western immunoblotting was used to quantity hTERT protein expression in all four OC cell lines. Treatment with metformin for 24 h significantly decreased the expression of hTERT mRNA (p<0.05-0.01) and protein in a dose-dependent manner in all of the OC cell lines (Figure 1C and 1D), suggesting that metformin may inhibit telomerase activity through rapidly decreasing hTERT expression.

### Effect of metformin on cell cycle progression and apoptosis

**Figure 2:**
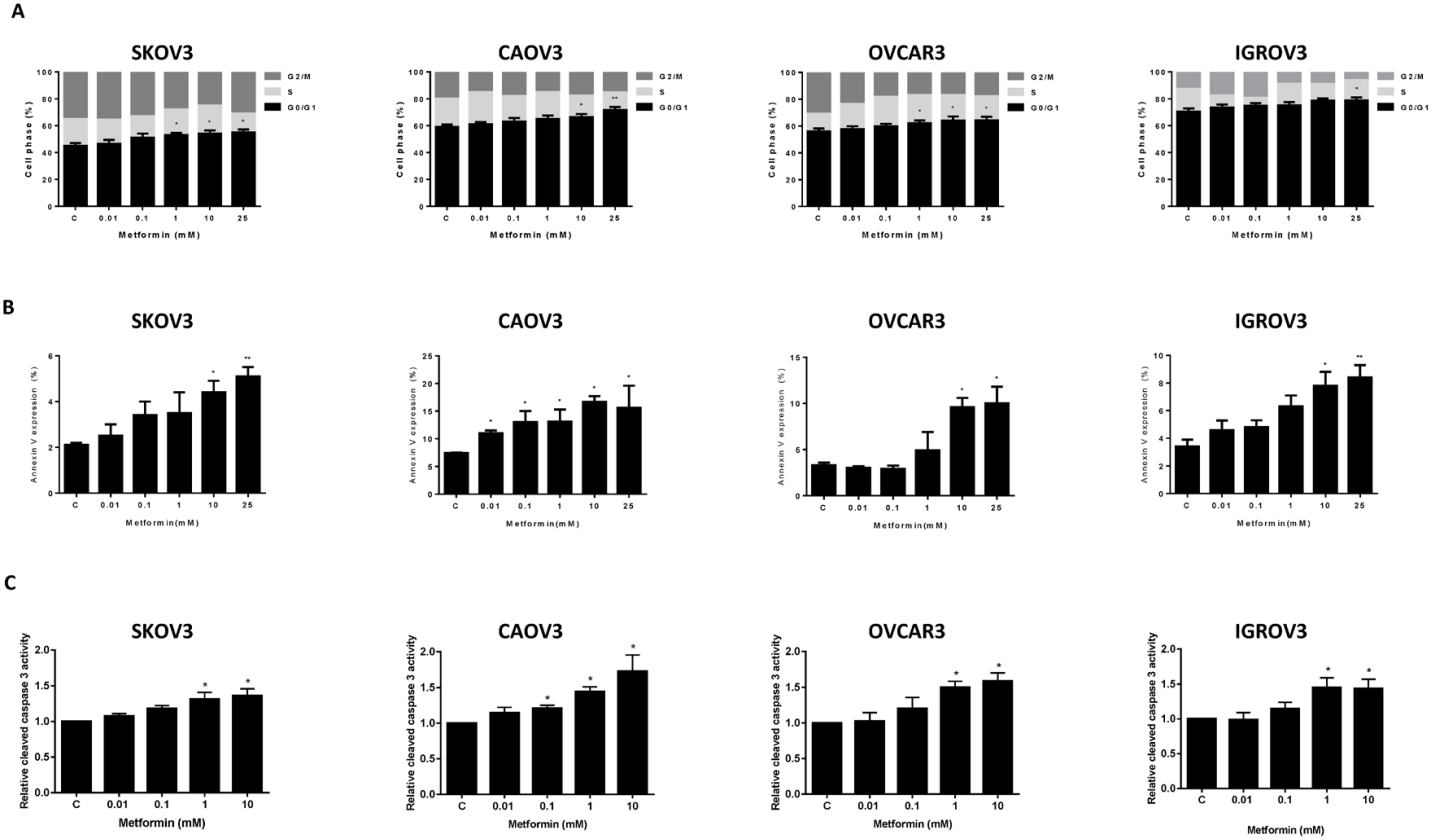
Metformin induced cell cycle G1 arrest and apoptosis The OC cell lines, SKOV3, CAOV3, OVCAR3 and IGROV1, were treated with metformin at the indicated concentrations for 36 h. Cell cycle analysis was performed by flow cytometry, and metformin was found to induce G1 arrest at dose of 1-25 mM **(A)**. Metformin increased Annexin V expression after 24 h of treatment **(B)**, and induced cleaved caspase 3 activity after 12 h treatment **(C)** in the OC cell lines. Results shown are representative of two independent experiments. *p<0.05, **p<0.01.

To evaluate the mechanism of growth inhibition by metformin, its effects on cell cycle progression and induction of apoptosis were analyzed after 36 h of treatment. As expected, metformin significantly induced G1 cell cycle arrest and reduced the number of cells in S phase in a dose-dependent manner in all four OC cells (p<0.05-0.01) (Figure 2A). To confirm whether the growth inhibition of ovarian cells *in vitro* was related in part to apoptosis, we evaluated the apoptotic effect of metformin on the OC cell lines by Annexin-V FITC stain analysis. This assay detects the phospholipid phosphatidylserine (PS) translocated from the inner (cytoplasmic) leaflet of the cell membrane to the external surface in very early apoptotic cells. As shown in Figure 2B, the percentage of apoptotic cells increased in a dose-dependent manner in the OC cell lines after 24 h of treatment with metformin (p<0.05).

In addition, we examined the effect of metformin on the activity of the caspase family of proteins to determine whether caspase activation contributes to metformin-induced OC cell apoptosis. Caspase 3, a specific marker for epithelial apoptosis, was assessed by ELISA assay. Metformin increased caspase 3 activity in the OC cell lines at doses of 1 and 10 mM (p<0.05) (Figure 2C).

### Effect of metformin on the AMPK and mTOR pathway

**Figure 3:**
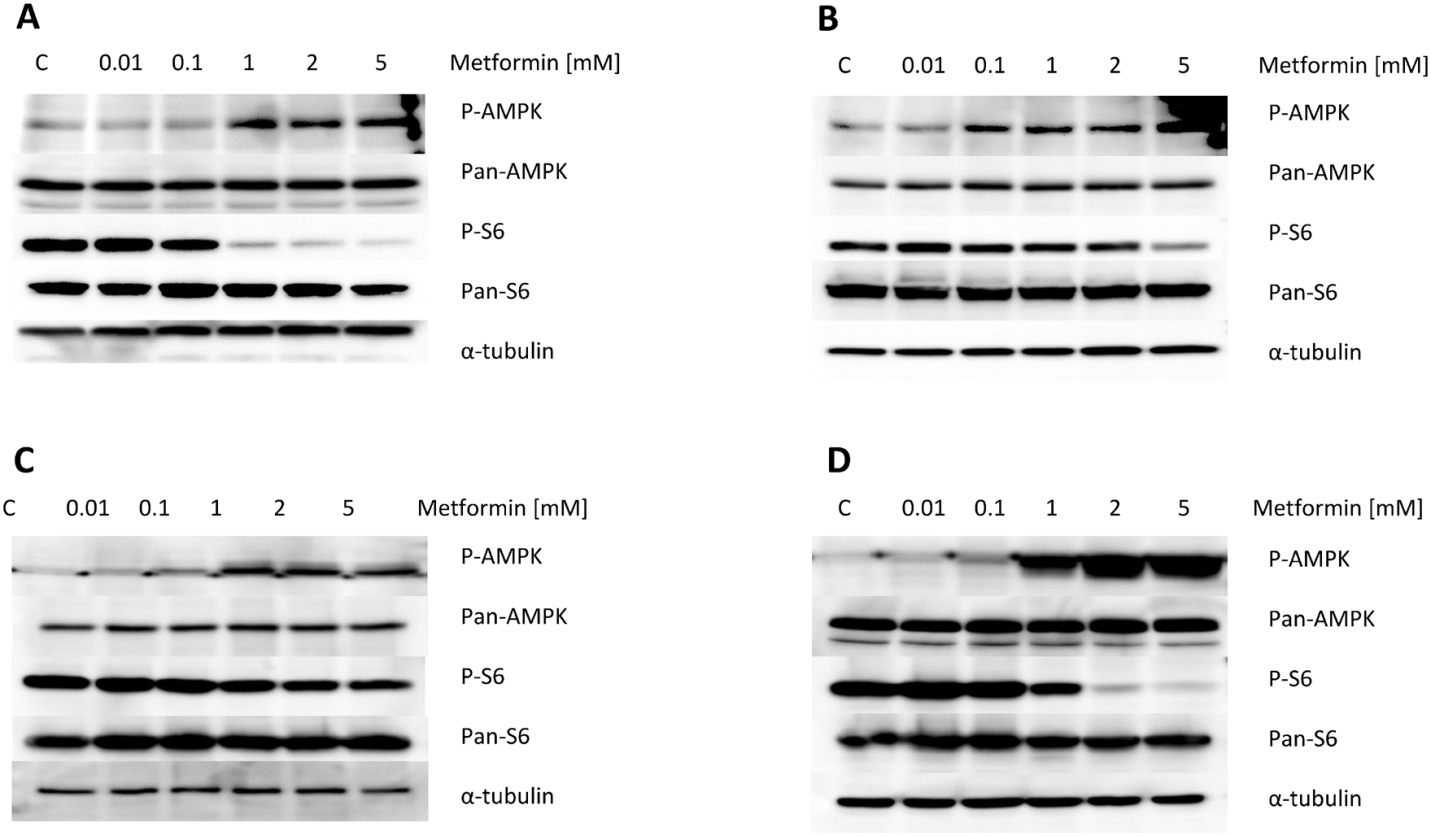
Metformin increased phosphorylation of AMPK and decreased phosphorylation of S6 in the OC cell lines The four OC cell lines were treated with metformin for 16 h. Protein was subsequently extracted, and western immunoblotting performed. Metformin induced expression of phosphorylated-AMPK and decreased expression of the phosphorylated-S6 protein. Results shown are one of three independent experiments.

To investigate the mechanisms underlying the inhibition of cell proliferation by metformin, we characterized the effect of metformin on relevant downstream signaling targets and pathways. Metformin induced phosphorylation of AMPK in a dose-dependent manner in all the OC cell lines, within 18 h of exposure (Figure 3A-3D). To further evaluate whether metformin affects the mTOR pathway *via* AMPK in the OC cells, we examined the phosphorylation of ribosomal protein S6, a downstream target of AMPK/mTOR. Metformin inhibited phosphorylation of S6 in a dose-dependent manner after 18 h of treatment in the OC cell lines, with the exception of the CAOV3 cells, for which metformin only inhibited phosphorylation of S6 at high doses (>5 mM, Figure 3B). Expression of pan-AMPK and pan-S6 was not affected by metformin. These findings suggest that metformin may exert its anti-tumor activity *via* activation of AMPK and subsequent inhibition of the mTOR pathway, resulting in decreased phosphorylation of S6.

### Effect of metformin on OC cell adhesion and invasion

**Figure 4:**
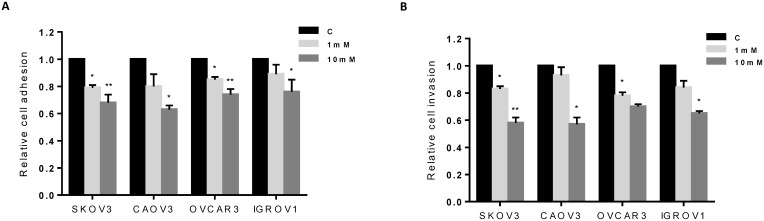
Metformin inhibited cell adhesion and invasion The four OC cell lines were treated with metformin at the indicated doses for 2 to 4 h. Adhesion was assessed by laminin-1 assay **(A)**, and invasion was detected by ChemoTx^®^ invasion assay **(B)**. Metformin inhibited adhesion and invasion in all four cell lines. The results are shown as the mean ± SD and are representative of three independent experiments. *p<0.05, **p<0.01.

Adhesion and invasion are believed to be important steps in OC metastasis. To evaluate the role of metformin on adhesion and invasion of OC cells, an *in vitro* adhesion assay and ChemoTx^®^ invasion assay were employed. Cell adhesion was decreased by 24-37% in all four OC cell lines after treatment with metformin *versus* vehicle at a dose of 10 mM for 2 h (p<0.05-0.01) (Figure 4A). In addition, metformin decreased cell invasion in all four cell lines after 4 h of treatment. At a dose of 10 mM, metformin reduced invasion by 30-42% compared to vehicle-treated groups (p<0.05-0.01) (Figure 4B). The inhibition of adhesion and invasion by metformin was dose-dependent for all cell lines tested. These results suggest that metformin may blunt OC cell adhesion and invasion, in addition to inducing cell cycle arrest and apoptosis.

### Effect of metformin on tumor growth in obese and lean KpB mice

**Figure 5:**
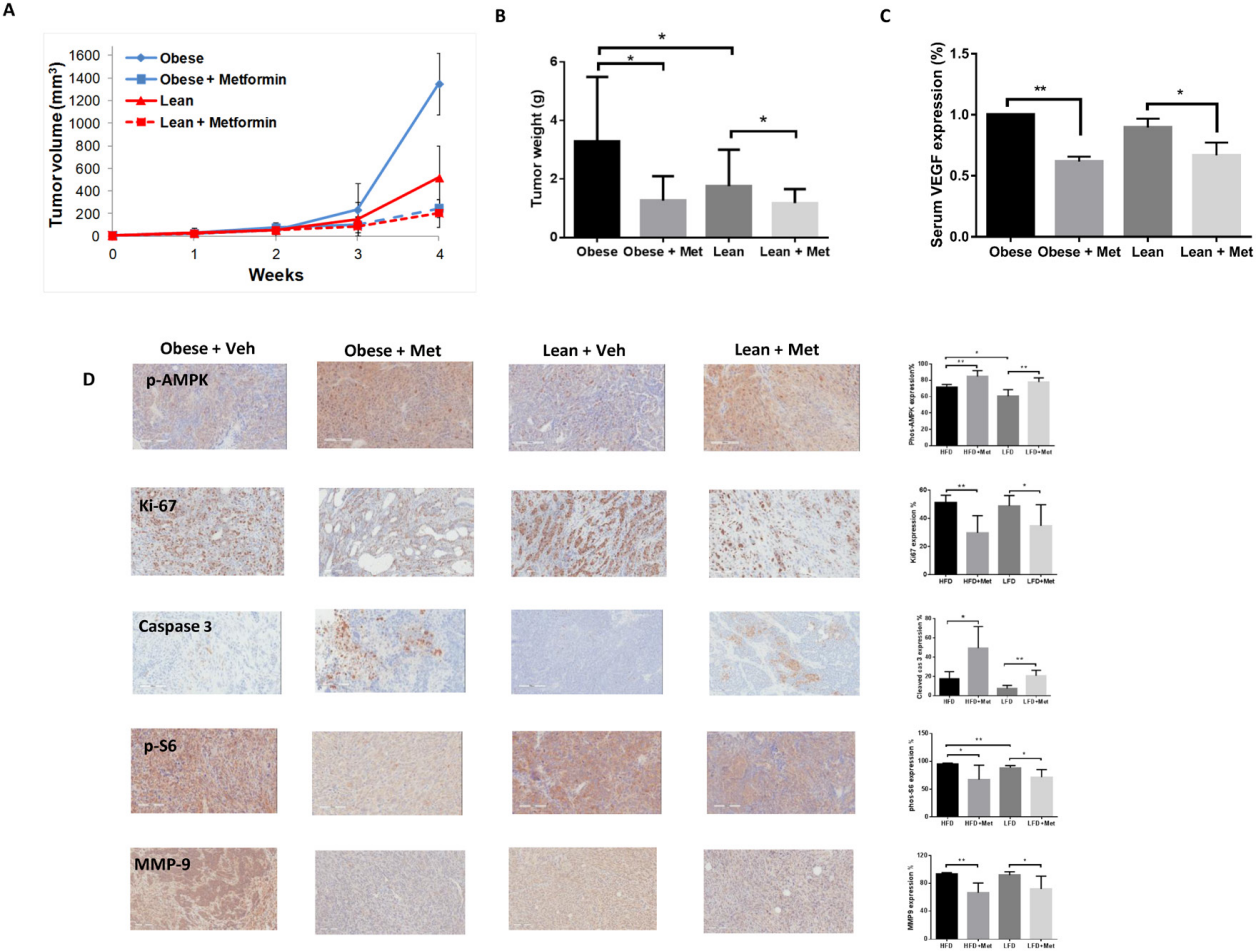
Metformin inhibited ovarian tumor growth in obese and lean KpB mice The KpB mice were fed a HFD (obese) or LFD (non-obese) starting at 3 weeks of age. Once a 1 cm ovarian tumor was palpated, mice underwent treatment with metformin (20 mg/kg oral gavage) *versus* vehicle for 4 weeks. Metformin significantly inhibited tumor volume and tumor weight **(A** and **B)**. Metformin reduced the level of serum VEGF in the HFD- and LFD-fed mice groups **(C)**. The effect of metformin on expression of Ki-67, phos-AMPK, phos-S6, cleaved caspase 3 and MMP-9 was assessed by immunohistochemistry **(D)**. *p<0.05, p**<0.01.

During the 4 weeks of metformin or vehicle treatment, the obese and lean KpB mice demonstrated tolerance of metformin and maintained normal activities. Regular twice-weekly measurements yielded no changes in blood glucose or body weight (data not shown) during treatment. At the time of sacrifice, the HFD-fed mice (obese) weighed a mean of 49.13 grams (gm) *versus* only 30.24 gm in the LFD-fed mice (lean) (p<0.01, data not shown). Tumor size was assessed by both tumor volume and tumor weight. Obesity significantly promoted tumor growth compared to lean mice group. Metformin inhibited tumor volume growth and weight in both the obese and lean mice after 4 weeks of treatment (Figure 5A and 5B). However, metformin-mediated decreases in tumor volume/weight in obese mice were significantly greater than in lean mice (60% *versus* 32%, respectively, p=0.003), suggesting that metformin’s anti-tumorigenic efficacy may be augmented in the obese state. ELISA assay showed that the production of VEGF in serum was significantly reduced following metformin treatment in both obese and lean KpB mice compared to the controls (p<0.01-0.05) (Figure 5C).

Immunohistochemical analysis was performed on the ovarian tumors after treatment with metformin or vehicle to assess effects on proliferation, apoptosis, and downstream targets of the mTOR pathway (Figure 5D). As compared to vehicle-treated mice, metformin decreased Ki-67, a marker of cell proliferation, and increased caspase-3, a marker of apoptosis, in the ovarian tumors of obese and lean KpB mice (p<0.05-0.01). In addition, metformin increased phosphorylation of AMPK (i.e. activating it) and decreased phosphorylation of S6, a downstream target of the mTOR pathway (p<0.05-0.01). Treatment with metformin also resulted in decreased expression of matrix metalloproteinase 9, a protein involved in the degradation of the extracellular matrix and subsequent invasion, in both the obese and lean KpB mice. Together, these *in vivo* findings in the KpB mouse model of OC support the *in vitro* findings in OC cell lines, suggesting that treatment with metformin decreases cell proliferation, induces apoptosis, reduces adhesion/invasion, activates AMPK and inhibits the mTOR pathway.

### Metabolic effects of metformin in the ovarian tumors of obese and lean mice

**Figure 6:**
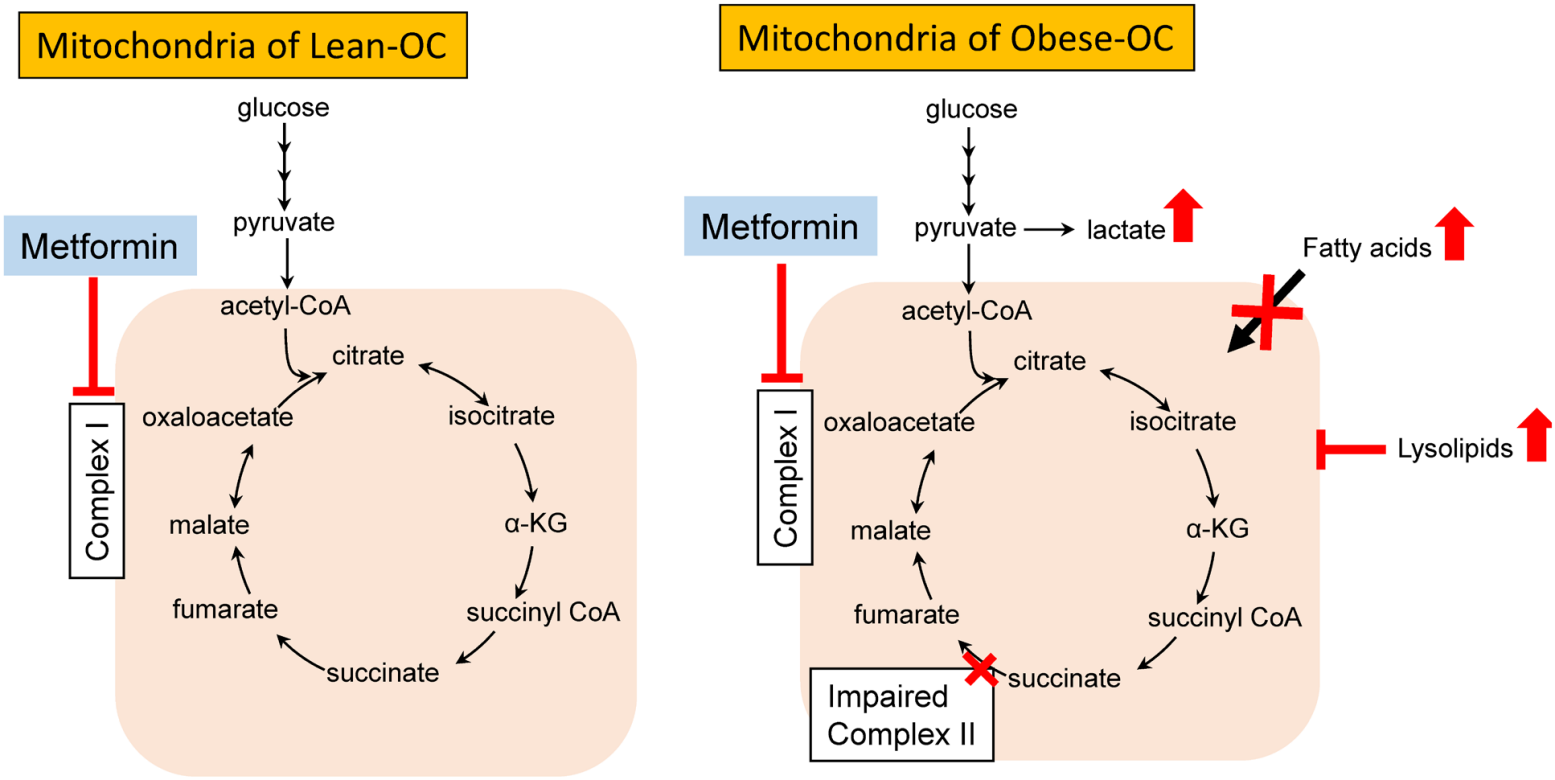
Schematic of metabolic changes in obese and lean endometrial tumors and impact of metformin treatment Metabolic pathways were dysfunctional in obese- *versus* lean-OCs in KpB mice, reflected by the inability to metabolize glucose, heightened omega-fatty acid oxidation for energy production and indications of impaired SDH/complex II. In the presence of metformin, complex I was inhibited in the OCs from both lean and obese KpB mice. However, when metformin inhibited complex 1 in the complex II impaired ovarian tumors in obese mice, this led to a profound switch in energy production from fatty acid oxidation to glycolysis. Lysolipids were also significantly increased in metformin-treated obese-OCs, leading to further disruption of mitochondrial function.

Metabolomic profiling revealed metabolic differences between the ovarian tumors from obese and lean KpB mice. 58 up- or down-regulated metabolites differentiated ovarian tumors in obese (obese-OCs) *versus* lean mice (lean-OCs) (Table 1 and Figure 6). Random Forest (RF) analysis distinguished an ovarian tumor as lean or obese with a predictive accuracy of 100%. Most strikingly, glucose levels were 3-fold higher in the ovarian tumors of the obese *versus* lean mice (p<0.05), and were accompanied by decreases in downstream intermediates of glycolysis, including pyruvate and lactate, indicating impaired glycolysis. Glutamine (1.7 fold) were also increased in the tumors from obese mice. Omega fatty oxidation appeared to be stimulated in the setting of obesity, as evidenced by decreases in n3 and n3 fatty acids (1.2 – 1.6 fold) and a 3-4 fold increase in several acyl-carnitines and dicarboxylic acids. Lysolipids were also significantly decreased in the ovarian tumors from obese *versus* lean mice (1.2 – 1.7 fold). Lastly, succinate levels were almost 5-fold higher in the ovarian tumors from obese *versus* lean mice, with a parallel decrease in fumarate and malate, indicating impaired succinate dehydrogenase (complex II) activity.

**Table 1:** Comparison of metabolic differences between the ovarian tumors from obese and lean KpB mice

Sub-Pathway	Biochemical Name	Obese/Lean*
**Glutamate** **Metabolism**	Glutamine	1.71
**Glycolysis**	Glucose	2.76
	Fructose-6-phosphate	0.52
	Isobar: F1, 6BP, G1, 6BP, myo-INS BPs	0.45
	Pyruvate	0.48
	Lactate	0.76
**TCA cycle**	Succinate	4.84
	Fumarate	0.62
	Malate	0.71
**Fatty Acid**	Palmitoylcarnitine	3.22
**Oxidation**	Stearoylcarnitine	4.41
	Oleoylcarnitine	4.45
	Azelate	4.55
	Undecanedioate	4.48
**n3 and n6**	Eicosapentaenoate	0.52
**Fatty Acids**	Docosapentaenoate	0.58
	Docosahexaenoate	0.81
	Dihomo-linolenate	0.73
	Arachidonate	0.61
	Adrenate	0.57
	Docosapentaenoate	0.43
	Docosadienoate	0.62
**Lysoplipids**	1-arachidonoylglycerophosphocholine	0.64
	1-palmitoylplasmenylethanolamine	**0.39**
	1-stearoylplasmenylethanolamine	**0.56**
	1-oleoylplasmenylethanolamine	**0.4**
	1-palmitoylglycerophosphoethanolamine	**0.41**
	1-stearoylglycerophosphoethanolamine	**0.62**
	2-stearoylglycerophosphoethanolamine	**0.46**
	1-oleoylglycerophosphoethanolamine	**0.53**
	1-linoleoylglycerophosphoethanolamine	0.68
	1-arachidonoylglycerophosphoethanolamine	**0.47**
	1-palmitoylglycerophosphoinositol	**0.39**
	1-stearoylglycerophosphoinositol	**0.55**
	1-oleoylglycerophosphoinositol	0.49
	1-linoleoylglycerophosphoinositol	0.33
	1-arachidonoylglycerophosphoinositol	**0.55**
	1-stearoylglycerophosphoserine	0.68
	1-oleoylglycerophosphoserine	0.77
	1-linoleoylglycerophosphoserine	**0.66**

*dark green and red = p<0.05, light green and pink = p<0.1.

Metabolic differences were also noted in the ovarian tumors from lean and obese KpB mice after metformin treatment (Table 2). Glucose levels were initially high within ovarian tumors of obese mice, falling with metformin treatment. Glycolysis was increased in the ovarian tumors of obese mice treated with metformin as compared to lean mice, as evidenced by elevations in glucose-6-phosphate (1.82 fold), fructose-6-phosphate (2.3 fold), fructose-1,6-bisphosphate (2.4 fold), 2,3-diphosphoglycerate (13.9 fold), dihydroxyacetone phosphate (2.44 fold) and most importantly, lactate (1.36 fold). Succinate levels decreased (1.8) while fumarate (1.6 fold) and malate (1.3 fold) increased in metformin-treated tumors from obese as compared to lean mice, suggesting a block in the conversion of malate to oxaloacetate (complex I activity). In addition, n3 and n6 fatty acids were increased with metformin treatment in obese- *versus* lean OCs (1.12 – 3.75 fold), indicating an inability to oxidize fatty acids when mitochondrial complex I and II are inhibited. In only the OCs of obese mice, metformin treatment resulted in elevations in lysolipids (2.2 – 4.7 fold). Glutamate also appeared to be oxidized in obese- as compared to lean-OCs, resulting in increases in α-ketoglutarate (1.53 fold), glucosamine-6-phosphate (1.8 fold) and N-acetylglucosamine-6-phosphate (2.13 fold).

**Table 2:** Comparison of metabolic changes with metformin treatment in the ovarian tumors from obese and lean KpB mice

Sub-Pathway	Biochemical Name	Lean-Met/Lean-Ctrl*	Obese-Met/Obese-Ctrl*
**Glycolysis**	Glucose	2.75	0.46
	Glucose-6-phosphate	0.91	1.82
	Fructose-6-phosphate	0.87	2.3
	Fructose-1-6-bisphosphate	0.68	2.4
	2,3-diphosphoglycerate	1.34	13.86
	Dihydroxyacetone phosphate	0.64	2.44
	Phosphoenolpyruvate	2.99	1.7
	Pyruvate	0.27	1.36
	Lactate	0.82	1.35
**TCA Cycle**	Citrate	0.46	0.94
	Alpha-ketoglutarate	1.23	1.53
	Succinylcarnitine	3.6	2.44
	Succinate	3.29	0.22
	Fumarate	0.73	1.57
	Malate	0.8	1.4
**Glutamine**	α-ketoglutarate	1.23	1.53
**Oxidation**	Glucosamine-6-phosphate	0.65	1.8
	N-acetylglucosamine-6-phosphate	0.56	2.13
**n3 and n6**	Eicosapentaenoate	0.54	1.12
**Fatty Acids**	Docosapentaenoate	0.48	1.94
	Docosahexaenoate	0.69	1.97
	Dihomo-linolenate	0.99	2.23
	Arachidonate	0.90	2.28
	Adrenate	0.51	2.1
	Docosapentaenoate	0.72	3.75
	Docosadienoate	0.57	1.83
**Lysolipids**	1-arachidonoylglycerophophocholine	1.5	2.6
	1-palmitoylplasmenylethanolamine	0.82	3.24
	1-stearoylplasmenylethanolamine	0.96	3.38
	1-oleoylplasmenylethanolamine	0.72	2.82
	1-palmitoylglycerophosphoethanolamine	0.87	3.0
	1-stearoylglycerophosphoethanolamine	0.72	2.21
	2-stearoylglycerophosphoethanolamine	0.57	2.68
	1-oleoylglycerophosphoethanolamine	1.18	2.72
	1-palmitoylglycerophosphoinositol	1.16	4.71
	1-stearoylglycerophosphoinositol	0.86	2.8
	1-arachidonoylglycerophosphoinositol	0.23	2.64
	1-stearoylglycerophosphoserine	2.15	2.5
	1-oleoylglycerophosphoserine	1.88	3.15

*dark green and red = p<0.05, light green and pink = p<0.1.

## DISCUSSION

In this study, we investigated the potential anti-tumorigenic activity of metformin in four human OC cell lines and a genetically engineered mouse model of serous OC under obese and lean conditions. In the OC cell lines, metformin was found to inhibit cellular proliferation *via* G1 phase cell cycle arrest, induce apoptosis, suppress hTERT mRNA expression and block cellular adhesion and invasion. Treatment with metformin resulted in the rapid activation of AMPK and decreased phosphorylation of ribosomal S6 kinase (S6K) in a dose-dependent manner, consistent with metformin’s known effect on mTOR pathway inhibition. In addition, we found that metformin had profound anti-tumorigenic effects in the KpB OC model; importantly, metformin was more efficacious in the tumors of obese (“obese-OC”) *versus* lean mice (“lean-OC”) that aligned with distinct metabolic effects depending on obesity status. Thus, our *in vitro* and *in vivo* studies support a potential role for metformin in OC, especially for obesity-drive disease.

Controlling energy metabolism is a fundamental requirement for cancer cells. AMPK is believed to be a key player in the regulation of energy metabolism [49, 54]. LKB1 and Ca^++^/calmodulin-dependent protein kinase kinase-B (CaMKKB) have been identified as two upstream kinases involved in regulating the activity of AMPK [55]. Activation of AMPK by metformin results in the regulation of multiple downstream signaling pathways involved in the control of protein, fatty acid and lipid synthesis, ultimately resulting in cancer cell growth inhibition through cell cycle arrest and apoptosis. AMPK’s immediate downstream targets include mTOR, which regulates S6 and 4EBP1, fatty-acid synthase (FAS), and p53/p21 [54]. Up-regulation of the PI3K/Akt/mTOR pathway has been documented in many cancers [56], including OC [28–34], and inhibition of this pathway is thought to be a promising therapeutic target for cancer treatment. Notably, numerous studies have shown that metformin significantly inhibits cancer cell proliferation and tumor growth in mouse models for many different cancers, including breast, colon, pancreatic, prostate, endometrial, and lung cancer, among others [36, 46, 57]. Our results confirm that metformin suppresses proliferation of OC cells in a dose–dependent manner *in vitro* (Figure 1) and inhibits tumor growth *in vivo* in the KpB mouse model, suggesting that the anti-tumor activity of metformin may have a therapeutic application in a broad spectrum of cancers, including OC.

Telomerase is composed of an RNA template (hTR) and the catalytic reverse transcriptase (hTERT), with hTERT acting as the rate-limiting determinant in the formation of functional telomerase [58]. In most normal, somatic cell types including normal ovarian tissues, telomerase activity is usually undetectable [59]. However, more than 90-97% of OCs express telomerase activity and hTERT mRNA expression [59]. Activation of telomerase and increased hTERT mRNA expression are thought to be markers of cell proliferation as well as represent a fundamental step in carcinogenesis in many cancers [58]. Since cancer and aging may share certain molecular processes, it is plausible that metformin may prevent and treat cancer by acting on the aging process including inhibition of telomerase activity [60, 61]. Our findings demonstrate for the first time that metformin inhibits hTERT mRNA in a dose-dependent manner in OC cells, indicating that hTERT may be another target for metformin in the inhibition of OC growth and potentially a sensitive biomarker for accessing the cellular response to metformin *in vivo* and *in vitro*. It is possible that this effect of metformin on hTERT is mediated through the mTOR pathway, as inhibition of AKT/mTOR has been shown to suppress telomerase activity and hTERT mRNA expression in a number of cancers [62, 63].

In OC, metastasis is believed to occur through cells detaching from the primary tumor and subsequently re-adhering to the intra-peritoneal cavity and invading across the basal lamina into the stroma. Invasion and metastasis are the leading causes for recurrence, poor prognosis and death in OC [64]. Adhesion and invasion are early steps involved in the metastatic process for OC, which has a complex molecular basis that likely involves adhesion molecules, cell surface receptors, oncogenes, chloride channels, fatty acid synthase and focal adhesion kinase [65–69]. Several studies have reported that metformin inhibits adhesion and invasion *in vitro* in a variety of different cancers through multiple cell singling pathways, such as NF-kB, MMP-2/9, AKT/ERK1/2, PKC and JUK/AP-1 [70–73]. Further supporting a link between metformin and metastatic potential, we found that metformin significantly inhibited adhesion and invasion in all four OC cells tested and led to a decrease in matrix metalloproteinase-9 (MMP-9) in ovarian tumors in the KpB mice, an enzyme intricately linked to extracellular matrix remodeling and angiogenesis.

Given that energy regulation is important in tumorigenesis and is altered by obesity, we evaluated the effects of metformin after first inducing obesity through dietary changes (HFD *versus* LFD) in the KpB mouse model. As seen in previous studies of metformin in OC mouse models [73–75], metformin inhibited tumor growth in the KpB mouse model. However, while metformin inhibited tumor growth in both the HFD- and LFD-fed mice, metformin had a more potent effect in the HFD-fed mice (60% decrease in HFD-fed mice, 32% decrease in LFD-fed mice). These results suggest that metformin may be a more beneficial therapeutic strategy in an obese *versus* lean host. Immunohistochemical analysis revealed that metformin decreased cell proliferation, induced apoptosis, and activated AMPK with subsequent downstream inhibition of S6 protein in the ovarian tumors of obese and lean mice, which is consistent with prior data [73] and our *in vitro* findings. Serum VEGF levels also decreased with metformin treatment in both obese and lean KpB mice, consistent with metformin’s known impact on angiogenic pathways. Overall, our findings are consistent with those demonstrated in a syngeneic OC model, whereas metformin and calorie restriction were both found to have greater impact on inhibition of tumor growth in HFD- *versus* LFD-fed mice [75]. Animal studies in other cancers have also evaluated the relationship between obesity and efficacy of metformin and found similar results to ours [76–78]. In particular, in both lung and breast cancer animal models, metformin was found to be more effective in decreasing tumor growth in animals fed a HFD compared to a low fat or standard diet [79, 80]. Furthermore, in a randomized, placebo-controlled pre-operative window study in breast cancer patients, women with higher body mass index (BMI) and Homeostatic Model Assessment of Insulin Resistance (HOMA) indexes had a greater response to metformin as evidenced by a decrease in Ki-67 staining [81]. These findings suggest that the anti-tumorigenic effects of metformin may be heightened in the setting of obesity and insulin resistance, due to its ability to improve the metabolic milieu of patients either indirectly or directly.

However, in striking contrast, metformin treatment has been found to elicit greater reductions in tumor growth in normoglycemic *versus* hyperglycemic conditions in a syngeneic OC mouse model [82], suggesting that metformin may have greater anti-tumorigenic efficacy in non-diabetic as opposed to diabetic patients. Taken together, these findings and ours emphasize that hyperglycemia and obesity are not interchangeable in their impact on modifying metformin response for cancer treatment. Further studies are needed to tease out the impact of high fat diet-induced *versus* hyperglycemia on metformin response in OC.

Metabolomic profiling profoundly delineated the effects of obesity on OC pathogenesis as well as suggested potential underlying mechanisms to metformin’s heightened efficacy in the setting of obesity (Figure 6). Metabolomic profiling revealed that glycolysis was preferentially stimulated in the ovarian tumors of obese mice treated with metformin as compared to lean mice, suggesting a switch in substrate from fatty acids to glucose. The lack of glycolysis in the more aggressive tumors in obesity was surprising, since the Warburg effect is typically associated with tumorigenesis [83]. However, recent reports suggest a more oxidative phenotype may prevail in more aggressive cancer models [84]. Indeed, as opposed to glucose, the more rapidly growing obese-OCs appeared to incompletely beta-oxidize fatty acids and switch to omega-fatty acid oxidation for ATP production and fueling growth as opposed to glucose, as evidenced by decreases in n3 and n6 fatty acids and corresponding increases in several acyl-carnitines and dicarboxylic acids. In addition, succinate levels rose and fumarate and malate levels fell in the ovarian tumors from obese *versus* lean mice, indicating impaired succinate dehydrogenase (complex II) activity. Mitochondrial dysfunction has been reported in tumors cells [85], including that of complex II in OC [86]. Lastly, lysolipids were markedly decreased in the obese- *versus* lean-OCs, suggesting that lysolipids were being re-acylated as a means to regenerate phospholipids for membranes biosynthesis and ultimately tumor growth. In summary, our findings suggest that obesity promotes alterations in tumor metabolomics that is associated with aggressive tumor behavior in the KpB OC mouse model.

Upon treatment with metformin, glucose levels fell and glycolysis was increased in the ovarian tumors of obese mice as compared to lean mice. Metformin is known to decrease mitochondrial respiration efficiency by inhibiting mitochondrial complex I, thus shifting the ATP production burden to anaerobic glycolysis [36, 46, 57]. In addition, succinate was depleted whereas fumarate and malate accumulated in metformin-treated tumors – but only in obese mice – a result consistent with restricted conversion of malate to oxaloacetate (complex I activity). These metabolic changes may underlie why obese-OCs have heightened susceptibility to metformin, i.e., obese-OCs have impaired mitochondrial complex II function that - when combined with metformin’s inhibition of complex I - leads to profound impairment of mitochondrial oxidative phosphorylation. Thus, the obese-OCs may become solely dependent on glycolysis for ATP production. Further supportive evidence is the dramatic rise in n3 and n6 fatty acids with metformin treatment in obese-OCs, indicating an inability to oxidize fatty acids when mitochondrial complex I and II are inhibited. In obese ovarian tumors, lysolipids were also increased with metformin treatment, suggesting that phospholipids were being degraded perhaps through stimulation of phospholipase A2 (PLA2) by metformin [87]. Lysolipids have detergent-like properties that permeabilize membranes [88], and thus may further disrupt mitochondrial function in the obese-OCs. Glutamate can also be oxidized to generate TCA cycle intermediates; this process was induced in the metformin-treated obese-OCs, possibly as a mechanism to overcome metformin’s inhibitory effects on mitochondrial metabolism.

These findings support our hypothesis that metformin may have differential direct metabolic effects to alter the established metabolic phenotype of ovarian tumors in obese *versus* lean mice, leading to improved efficacy in treating tumors which develop in an obese host environment. Obese-OCs appear more reliant on fatty acid oxidation as opposed to glycolysis for ATP production, with coincident impaired mitochondrial complex II function. In contrast, the opposite was true for lean-OCs. Given that the effects of metformin to inhibit the mTOR pathway were similar between ovarian tumors from obese and lean mice, we postulate that metformin’s direct metabolic effects on inhibition of mitochondrial complex I drive the increased response in complex II-impaired tumors from obese mice (Figure 6). Clinical trials are already underway for metformin in OC patients [89], and our findings underscore the importance of evaluating the metabolic milieu of a patient and their corresponding tumor as potential biomarkers of metformin response in cancer therapeutic trials.

## MATERIALS AND METHODS

### Cell culture and reagents

Four OC cell lines, SKOV3, IGROV1, CAOV3 and OVCAR3, were used for these experiments. SKOV3 cells were grown in DMEM/F12 supplemented with 10% fetal bovine serum (FBS), 100 units/ml penicillin and 100 μg/ml streptomycin under 5% CO_2_. IGROV1, CAOV3 and OVCAR3 cells were maintained in RPMI 1640 containing 10% FBS, 100 units/ml penicillin and 100 ug/ml streptomycin. Metformin, MTT dye, RNase A and anti-α-tubulin antibody were purchased from Sigma (St. Louis, MO). The anti-phosphorylated-AMPK, anti-pan-AMPK, anti-phosphorylated-S6 and anti-pan-S6 antibodies as well as the caspase-3 ELISA kit were purchased from Cell Signaling (Beverly, MA). The Annexin V FITC Kit was purchased from BioVison (Mountain View, CA). The ChemoTx^®^ Invasion Kit was from NeuroProbe (Gaithersburg, MD). Enhanced chemiluminescence Western blotting detection reagents were purchased from Amersham (Arlington Heights, IL). All other chemicals were purchased from Sigma.

### Cell proliferation assay

The SKOV3, IGROV1, CAOV3 and OVCAR3 cells were plated and grown in 96-well plates at a concentration of 4000 to 6000 cells/well for 24 hours (h). Cells were then treated with varying doses of metformin for 72 h. Viable cell densities were determined by metabolic conversion of the dye MTT. MTT (5 mg/ml) was added to the 96-well plates at 10 μl/well, and the plates were then incubated for an additional 1-2 h. The MTT reaction was terminated by the addition of 100 ul DMSO. The MTT assay results were read by measuring absorption at 595 nm. The effect of metformin was calculated as a percentage of control cell growth obtained from PBS (0.1%) treated cells grown in the same 96-well plates. Each experiment was performed in triplicate and repeated three times to assess for consistency of results.

### Mitochondrial complex I activity

Mitochondrial complex I activity was measured using the Complex I Activity K Assay kit from MitoSciences (Eugene, OR), according to the manufacturer’s protocol. Briefly, protein was extracted from six well culture plates treated with metformin or vehicle by adding the provided detergent solution to each well. 50 ug of protein was used to determine the activity of complex I. After loading the proteins onto 96 well plates coated with an anti-complex I monoclonal antibody, the plates were incubated for 3 h at room temperature. Optical Density (OD450 nm) was measured using a Tecan plate reader in kinetic mode at room temperature for 30 minutes (min).

### Flow cytometry

The OC cell lines were plated at 2.5-3.5 x 10^5^ cells/well in 6-well plates in their corresponding media for 24 h. Subsequently, the cells were treated with metformin at varying concentrations for 36 h. Cells were collected, washed twice with PBS, fixed in a 90% methanol solution and then stored at -20°C until flow cytometric analysis was performed. On the day of analysis, cells were washed and centrifuged twice using cold PBS, suspended in 100 μl PBS and 10 μl of RNase A solution (250 ug/ml), followed by incubation for 30 min at 37°C. After incubation, 110 μl of PI (100 ug/ml) stain was added to each tube and incubated at 4°C for at least 30 min prior to analysis. Flow cytometric analysis was performed on a CyAn machine (Beckman Coulter, Miami, FL). ModFit (Verity Software House, Topsham, ME) was utilized for the analysis to control for dead cells and debris. All experiments were performed in triplicate and repeated twice to assess for consistency of response.

### Apoptosis assay for caspase 3

The four OC cell lines were cultured in 6-well plates at 2-4 x 10^5^ cells/well for 24 h and then treated with metformin at various doses in 0.5% stripped serum for an additional 24 h. ELISA analysis with a Caspase-3 kit was performed according to the manufacturer instructions. Briefly, the cells were lysed, and protein concentrations measured to confirm equal loading onto an ELISA plate. Reagents were added as described by the manufacturer, and the ELISA plate was read by measuring absorption at 450 nm. All experiments were performed in triplicate and repeated twice to assess for consistency of response.

### Annexin V assay

Annexin V was assessed using the Annexin V-FITC Apoptosis Detection Kit. Briefly, the OC cell lines were plated at 3 x 10^5^ cells/well in 6-well plates for 24 h, and then treated with metfromin at the indicated concentrations for 24 h. Cells were collected, washed with PBS and resuspended in the binding buffer. 5 μl of annexin V-FITC and 5 μl of propidum iodide (PI, 50 μg/ml) were added in the binding buffer for 5 min in the dark. The samples were immediately measured by BD FacsCalibur flow cytometer (BD Biosciences, USA). The results were analyzed by Cellquest software. Apoptotic cells were expressed as a percentage of the total number of stained cells counted.

### Real-time RT-PCR for hTERT

Total RNA was extracted using the RNAqueos kit (Ambion, Austin, TX) and further purified by the DNA-free kit (Ambion). The reverse transcription and PCR reactions were performed using the TaqMan Gold one-step RT-PCR kit in the ABI Prism 7700 Sequence Detection System (Applied Biosystems, Foster City, CA). Reverse transcription was carried out at 48°C for 30 min. The PCR conditions consisted of a 10 minute step at 95°C, 40 cycles at 95°C for 15 seconds each and 1 minute at 65°C. A housekeeping control gene, acidic ribosomal phosphoprotein P0 (RPLP0, also known as 36B4), was used as an internal control to correct for differences in the amount of RNA in each sample. Primers and fluorogenic probes for hTERT and RPLP0 have been described previously [90]. The standard curve for hTERT was generated by using dilutions of a known amount of cRNA synthesized by *in vitro* transcription of a cloned fragment. The normalized level of hTERT in each sample was estimated by a ratio of the hTERT level to the RPLP0 level, as described previously [90]. Each experiment was performed in triplicate and repeated twice to assess for consistency of results.

### Western immunoblotting

The SKOV3, IGROV1, CAOV3 and OVCAR3 cells were plated at 2-4 x 10^5^ cells/well in 6-well plates in their corresponding media and then treated for 18 h with metformin in 0.5% stripped serum. Cell lysates were prepared in RIPA buffer (1% NP40, 0.5 sodium deoxycholate and 0.1% SDS) plus PhosStop. Equal amounts of protein were separated by gel electrophoresis and transferred onto a nitrocellulose membrane. The membrane was blocked with 5% nonfat dry milk and then incubated with a 1:1000 dilution of primary antibody overnight at 4°C. The membrane was then washed and incubated with a secondary peroxidase-conjugated antibody for 1 h after washing. Antibody binding was detected using an enhanced chemiluminescence detection buffer and the Alpha Innotech imaging system (San Leandro, CA). After developing, the membrane was stripped and re-probed using antibodies against pan-S6, pan-AMPK and α-tubulin. Each experiment was repeated three times to assess for consistency of results.

### Measurement of VEGF levels

To measure the vascular endothelial growth factor (VEGF) concentration in the serum of mice after exposure to metformin, 10 μl of serum was analyzed using a VEGF ELISA kit (R&D Systems, Minneapolis, MN), according to the manufacturer’s instructions. The optical density at 570 nm of each well was measured using a Tecan reader (Morrisville, NC).

### Adhesion assay

Each well in a 96-well plate was coated with 100 μl laminin-1 and incubated at 37°C for 1 h. 200 μl blocking buffer was then added to each well for 45-60 min at 37°C. The wells were then washed with PBS and the plate was allowed to chill on ice. To each well, 2.5 x 10^3^ cells were added with PBS and varying concentrations of metformin directly. The plate and cell/treatment suspension was then allowed to incubate at 37°C for 2 h. The medium was then aspirated, and the cells were fixed by directly adding 100 μl of 5% glutaraldehyde and incubating for 30 min at room temperature. Adhered cells were then washed with PBS and stained with 100 μl of 0.1% crystal violet for 30 min. The cells were then washed repeatedly with water, and 100 μl of 10% acetic acid was added to each well to solubilize the dye. After 5 min of shaking, the absorbance was measured at 570 nm using a FLUOstar OMEGA plate reader from BMG Labtech (Cary, NC).

### Invasion assay

Invasion was assessed with the ChemoTx^®^ invasion kit (Gaithersburg, MD). Briefly, cells were starved in serum-free medium for 24 h. Cells were then collected, washed and resuspended in Gey's Balanced Salt Solution + 1% BSA with varying concentrations of metformin. To each well in a 96-well plate, 299 μl of media was added, along with varying concentrations of metformin. The framed filter membrane was carefully fitted to the top of the plate. The plate was allowed to incubate at 37°C for 4 h to allow for invasion into the lower compartment. These cells were then stained with 3 μl of MTT (5 mg/mL in RPMI-1640) and allowed to incubate at 37°C for 1 h. This liquid was then aspirated, the wells were washed with PBS, and the MTT dye was solubilized using 20 μl of DMSO. The absorbance was then measured at 595 nm using a FLUOstar OMEGA plate reader from BMG Labtech (Cary, NC).

### Dietary exposures and metformin treatment in the KpB mouse model

For our *in vivo* studies, we used the K18-gT_121_^+/-^; p53^fl/fl^; Brca1^fl/fl^ (KpB) mouse genetically engineered mouse model of serous epithelial OC (generously supplied by Terry Van Dyke, PhD, NIH) in which there is somatic deletion of Brca1 and p53 and inactivation of the retinoblastoma proteins *via* injection of an adenoviral vector expression Cre [91]. All experimental animals were maintained in accordance with the Institutional Animal Care and Use Committee (IACUC) and the NIH guide for the Care and Use of Laboratory Animals. OC generation in obese and lean KpB mice was conducted as previously described [35]. Three week-old female KpB mice were randomly divided into four treatment groups (n=10 per group). To mimic diet-induced obesity (DIO), half of the mice were subjected to a high fat diet (HFD), in which 60% of calories were derived from fat, while the other half were subjected to a low fat diet (LFD), in which only 10% of calories were derived from fat (Research Diets, New Brunswick, USA). The HFD and LFD were started at 3 weeks of age. At 6 weeks of age, the mice were anesthetized with ketamine, and the right ovary was exposed. AdCre was delivered into the right ovary using an injection of 5 μl of AdCre virus into the ovarian bursa with a Hamilton syringe and a 30-gauge beveled needle under the control of a dissection microscope. The recombinant adenovirus Ad5-CMV-Cre (AdCre) was purchased from the University of Iowa Transfer Vector Core at a titre of 10^11^-10^12^ infectious particles/ml.

Mice were maintained on either the LFD or HFD and were monitored weekly by palpation for the appearance of tumors. Once a 0.1 cm ovarian tumor was palpated, HFD-fed and LFD-fed mice initiated treatment with either metformin (200 mg/kg, oral gavage) or vehicle (PBS), and treatment continued for 4 weeks. Tumor size was checked twice a week using palpation until tumors had grown to a size amenable to caliper measurement. Tumor volume was calculated using the following equation: volume (mm^3^) = (*a* x *b*^2^)/2, where *a* is the largest diameter and *b* is the smallest diameter. Animals were weighed weekly throughout the study. At sacrifice, mice and tumors were weighed and blood samples were taken. Half of the tumor was snap-frozen and stored at ^_^80°C, and the other half was fixed in 10% neutral-buffered formalin and paraffin embedded. Pathologic evaluation of histologic findings was carried out by a board certified pathologist according to existing human epithelial OC classifications.

### Immunohistochemistry

Five micrometer paraffin sections, prepared from the KpB mice, were used for immunohistochemical (IHC) analysis. Staining procedures were performed at the IHC Animal Core Facility at the University of North Carolina. The following primary antibodies were used: Ki-67 (Cell Signaling, 1:800), MMP-9 (Santa Cruz, 1:500), cleaved caspase-3 (Cell Signaling, 1:100), phosophorylated-AMPK and phosphorylated S-6 protein (Cell Signaling, 1:1000). Further processing was carried out using ABC-Staining Kits (Vector Labs, Burlingame, CA) and hematoxylin. IHC slides were scanned, analyzed, and scored by Aperio and ImageScope software (Vista, CA).

### Metabolomic profiling

Metabolomic profiling was performed on ovarian tumors obtained from obese and lean KpB mice treated with either vehicle or metformin. Samples were analyzed by Metabolon (Research Triangle Park, NC) according to their standard protocols 7 [92–95] and our previous work [96]. Briefly, unbiased global metabolomic profiling was achieved using methanol extracts of tumor tissues normalized to tissue weight. Analysis of extracts consisted of either ultrahigh performance liquid chromatography (Waters Corporation, Milford, MA) coupled with tandem mass spectrometry (UHPLC/MS/MS; Thermo-Finnigan, San Jose CA) in positive and negative ionization modes, or *via* gas chromatography/MS analysis (Thermo-Finnigan). Metabolites in tumor tissues were positively identified by matching chromatographic retention time, mass and MS/MS fragmentation patterns to a reference library of over 2500 purified, authenticated biochemicals. Data are presented as relative measures of “scaled intensity” and median scaling to 1. Missing values were imputed with the minimum.

### Statistical analysis

Data are presented as mean ± S.E.M. Statistical analysis of the differences between groups was determined using the two-sided unpaired Student’s *t*-test using GraphPad software (La Jolla, CA), and a value of p<0.05 was considered statistically significant. The non-parametric ANOVA was used to test if there were differences in metformin’s effects in obese *versus* lean KpB mice.

For the metabolomic profiling, two types of statistical analyses were performed: (1) significance tests and (2) classification analysis. For pair-wise comparisons, Welch’s t-tests and/or Wilcoxon’s rank sum tests were performed. Where appropriate, repeated measures analysis of variance (ANOVA) was used. For classification analysis, random forest analyses were performed. Random forest is a supervised classification technique based on an ensemble of decision trees [97]. For a given decision tree, a random subset of the data with identifying true class information is selected to build the tree (“bootstrap sample” or “training set”), and then the remaining data, the “out-of-bag” (OOB) variables, are passed down the tree to obtain a class prediction for each sample. This process is repeated thousands of times to produce the forest. The final classification of each sample is determined by computing the class prediction frequency (“votes”) for the OOB variables over the whole forest. Statistical analyses were performed with the program “R” (http://cran.r-project.org/).
